# Aging, Economic Hardship, and Depression Among Ethnic Groups in Bangladesh: A Cross‐Sectional Study

**DOI:** 10.1002/hsr2.71732

**Published:** 2026-01-08

**Authors:** Kanis Fatama Ferdushi, Al Mahmud, Sabina Islam, S.M. Saydur Rahman

**Affiliations:** ^1^ Department of Statistics Shahjalal University of Science and Technology Sylhet Bangladesh; ^2^ School of Dental Sciences Universiti Sains Malaysia Kubang Kerian Kelantan Malaysia; ^3^ Department of Mathematics Shahjalal University of Science and Technology Sylhet Bangladesh

**Keywords:** aging, Bangladesh, depression, economic hardship, ethnic

## Abstract

**Background and Aims:**

Depression is a major public health issue, particularly among elderly populations, and is influenced by sociodemographic, health, and lifestyle factors. Ethnic variations in depression remain underexplored in Bangladesh. This study aimed to determine the prevalence of depression among elderly individuals from different ethnic groups and to identify associated risk factors.

**Methods:**

This cross‐sectional study was conducted among 632 respondents aged 55 years and above in Rangamati, Sylhet, and Mymensingh, Bangladesh. Depression symptoms were assessed using the validated Patient Health Questionnaire (PHQ‐9). Respondents were categorized as “not depressed” (score 0–9) and “depressed” (score ≥ 10). Data were collected using a structured questionnaire on sociodemographic, economic, and lifestyle factors. Descriptive statistics and logistic regression were used to estimate odds ratios (OR) with 95% confidence intervals (CI) at a significance level of *p* < 0.05. Analyses were performed in Stata 14.2.

**Results:**

The prevalence of depression was 26.6% (168/632). The mean age of participants was 59.73 years (SD = 8.08). Older participants had higher odds of depression: aged 60–70 years (OR = 1.25, 95% CI: 1.01–1.56, *p* < 0.05) and aged ≥ 71 years (OR = 2.85, 95% CI: 2.15–3.79, *p* < 0.001) compared to those aged < 60 years. Ethnic differences observable Chakma (43.45%, 73/632), Manipuri (41.67%, 70/632), and Garo (14.88%, 25/632) showed significant variation (*p* < 0.001). Longstanding health problems increased depression risk (OR = 6.47, 95% CI: 4.12–9.82, *p* < 0.001). Hypertension, frequent doctor visits, and poor wealth were also associated with depression. Dietary factors, such as sugar, alcohol, and red meat, showed exploratory associations.

**Conclusions:**

Depression is prevalent among elderly populations in Bangladesh, with significant variation across ethnic groups. Age, chronic illness, and socioeconomic disadvantage were key predictors. Findings highlight the need for culturally sensitive mental health interventions and targeted support for elderly minorities and those with chronic conditions.

## 1 Introduction

Depression is a serious problem, and major depressive disorder (MDD) is one of the most common and costly mental health conditions. It is expected to become the world's biggest health burden, making it important to understand its causes [1, 2]. With emotional dysfunction increasingly recognized as central over the past two decades, driving extensive research [3]. The age category played a crucial role in determining mental health. Older adulthood has a transitional phase characterized by physical aging and challenges that impact mental and social well‐being. As the brain undergoes natural aging, along with declining physical health and potential cerebral conditions, the prevalence of mental and behavioral disorders generally rises with age [4]. Among the different mental disorders, depression imposes the highest burden on the elderly. It reduces their quality of life and increases dependence on others. Untreated depression can lead to serious clinical and social consequences in their lives [5].

Depression is notably common among adolescents in Bangladesh, with girls exhibiting higher prevalence across various sociodemographic, lifestyle, and anthropometric groups. Several sociodemographic and lifestyle factors are linked to depression in this age group [6]. Many Bangladeshi undergraduate admission candidates face clinical depression or are at risk. Factors like admission confidence, study duration, blackmail, relationships, academic performance, family conflicts, and religiosity impact their mental health [7]. In the prior study among university students, around half of the students showed symptoms of depression. Key contributing factors included losing a study year, poor academic performance, drug exposure, personal or family history of depression, and excessive social media use [8].

Bangladesh is experiencing a steady demographic shift, with the proportion of older adults increasing as life expectancy continues to rise. Aging often brings multiple challenges, including declining physical health, reduced income, and limited access to healthcare and social support systems. Among the country's diverse population, ethnic minority groups, many of whom reside in remote or disadvantaged areas, are particularly vulnerable due to their socioeconomic marginalization and limited access to essential services. Bangladesh is home to numerous indigenous communities.

Bangladesh is home to a rich mosaic of indigenous communities, each with distinct languages, cultural traditions, and social structures that contribute to the nation's cultural diversity. These groups are primarily concentrated in the Chittagong Hill Tracts and various northern and northeastern regions of the country, including Rangamati, Bandarban, Khagrachari, Mymensingh, and Sylhet. Despite their longstanding presence and unique cultural heritage, many indigenous communities remain socially and economically marginalized, often facing challenges related to land dispossession, limited access to education and healthcare, and underrepresentation in national policy frameworks. Their traditional livelihoods, such as shifting cultivation, weaving, and small‐scale farming, are increasingly threatened by environmental changes and development pressures. Recognizing and understanding the well‐being of these indigenous populations, particularly their mental health and aging experiences, is therefore essential for promoting inclusive and equitable development in Bangladesh.

According to statistics, the estimated number of these communities varies due to challenges in defining them. Generally, estimates range from the 27 ethnic groups recorded in the 2011 census to the 59 indigenous peoples [9]. Mental health coverage in the region is largely neglected due to geographical challenges and political reluctance stemming from ethnic conflicts between Bangladeshi settlers and indigenous communities. Additionally, existing mental health services fail to consider indigenous cultural characteristics, such as language and values, leading to reluctance to cross care [10, 11]. However, this study addresses the overlooked issue of ethnic disparities in depression among aging populations in Bangladesh. By examining prevalence, risk factors, and socio‐cultural influences, the research aims to enhance understanding and support the development of culturally tailored mental health interventions.

## 2 Methodology

### 2.1 Study Design and Setting

This cross‐sectional study was carried out from July 2022 to June 2023 across three districts in Bangladesh: Rangamati, Sylhet, and Mymensingh. These locations were selected to ensure representation of diverse ethnic groups, specifically the Chakma, Manipuri, and Garo communities, who predominantly reside in these areas. The regions were chosen based on their distinct socio‐cultural attributes and the recognized health challenges experienced by older adults within these communities.

### 2.2 Study Population and Sampling

**Figure 1:**
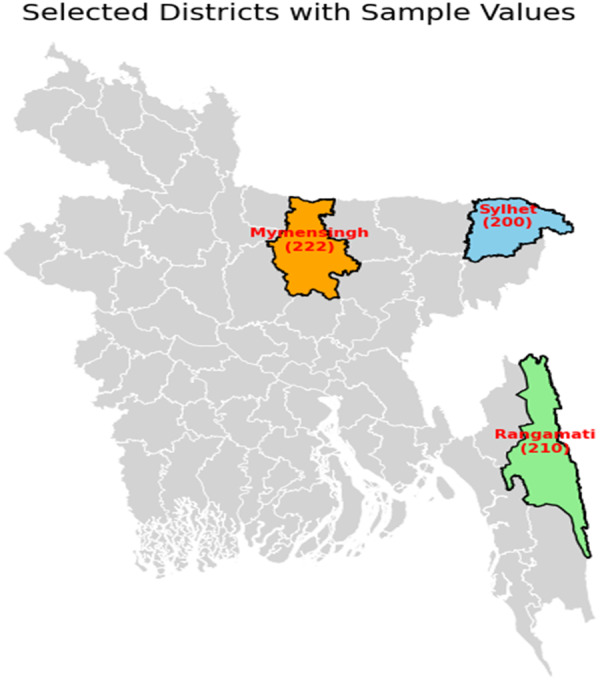
The study areas of Bangladesh. This map illustrates the geographic locations of the three selected districts—Rangamati, Sylhet, and Mymensingh—which represent the study regions. Each district is distinctly highlighted with its corresponding sample size: Rangamati (210), Sylhet (200), and Mymensingh (222). Other districts are shown in light gray for contextual reference.

This study focused on individuals aged 55 years and older from the Rangamati, Sylhet, and Mymensingh districts. Participants were required to meet the following inclusion criteria: being 55 years or older and willing to undergo a general health assessment and interview. A total of 632 individuals were selected using simple random sampling, with proportional representation from the three districts to ensure adequate inclusion of the Chakma, Manipuri, and Garo ethnic groups. Eligible individuals from selected households were invited to take part in the study (Figure 1). All participants were unrelated individuals, and no family or kinship relationship existed among the selected cases.

### 2.3 Data Collection

To investigate various health issues and conditions among the participants, a survey design incorporating multiple indicators was utilized. The study adhered to the standards set forth by the Helsinki Declaration of 2000 (revised version), and written informed consent was obtained from each participant before collecting any data [12]. After receiving formal consent (both written and verbal), each participant's health profile was obtained through a general health assessment and a structured questionnaire. This questionnaire included questions related to self‐reported health problems, food habits, daily activities, and socio‐demographic information.

### 2.4 Inclusion and Exclusion Criteria

The inclusion criteria were adults aged 55 years and above who were permanent residents of Rangamati, Sylhet, and Mymensingh districts, representing the Chakma, Manipuri, and Garo ethnic groups. Individuals were included if they were able to communicate and provide informed consent. The exclusion criteria included respondents who were below 55 years, had severe cognitive impairment, or were unable to complete the interview due to illness or communication difficulties. This study specifically focused on older adults because this population is at increased risk of depression due to age‐related health and social challenges. Therefore, the findings are interpreted within the context of this age group rather than generalized to the entire population.

### 2.5 Explanatory Variables

This study explores the relationship between depression and various socioeconomic, health, and dietary factors among the Chakma, Manipuri, and Garo ethnic groups in Bangladesh. The socioeconomic factors include demographics (gender, age, religion, and ethnicity), financial aspects (income, wealth, and financial support), and lifestyle indicators (occupation, education, marital status, body mass index, family behavior, and hospital visits). Health‐related factors cover current health conditions, chronic illnesses (diabetes and hypertension), and healthcare access (consultations and doctor visits). Dietary habits include meal frequency, consumption of meat, eggs, fruits, vegetables, sugar, oil, soft drinks, dairy products, alcohol, sleep quality, and outdoor activities.

### 2.6 Outcome Variable

Depressive symptoms were assessed using the Patient Health Questionnaire (PHQ‐9) (see Table S1), a widely used screening tool for measuring depressive symptoms that has been applied in numerous studies across Bangladesh [13, 14]. The dependent variable, “Depression,” was categorized into two groups: “Not depressed” (PHQ‐9 score between 0 and 9) and “Depressed” (PHQ‐9 score above 9). It should be noted that the PHQ‐9 is a screening instrument rather than a diagnostic tool, and therefore, the outcomes reflect symptom severity rather than clinical diagnosis.

### 2.7 Data Reliability and Validity

To ensure the reliability and validity of the data, several methods were employed in this study: (i) examination of data response errors, (ii) descriptive analysis to assess variability, (iii) graphical representation for visual analysis, and (iv) application of statistical techniques, including the chi‐square test, logistic regression, Hosmer−Lemeshow, and adjusted receiver operating characteristic (ROC) curve.

### 2.8 Statistical Analysis

Statistical analyses were performed using Stata version 14.2 (StataCorp LP, College Station, TX, USA). A *p* < 0.05 was considered statistically significant at 5% level of significance. All tests were two‐sided, and results are presented as odds ratios (ORs) with 95% confidence intervals (CIs). Descriptive statistics were summarized using means and standard deviations (SDs) for normally distributed data. Associations between categorical variables were initially examined using the chi‐square test, followed by multivariable logistic regression to identify independent predictors of depression [15]. The primary analyses were prespecified to assess the relationship between depression and sociodemographic, economic, and lifestyle factors, while subgroup analyses by ethnicity were considered as exploratory. The analysis and reporting followed the Statistical Analyses and Methods in the Published Literature (SAMPL) guidelines [16] and recommendations for reporting clinical research [17].

### 2.9 Model Selection

The final model was constructed in three phases. Initially, a univariate analysis was performed to assess the association between each independent variable and the outcome variable, selecting those with a *p* < 0.2. In the next phase, only statistically significant variables were incorporated into a preliminary multivariable model. Lastly, the significant variables from this preliminary model were retained to develop the final multivariable logistic regression model, as summarized in the outcome table.

### 2.10 Model Performance

The model's performance was assessed using various metrics, including the ROC curve, Hosmer−Lemeshow goodness‐of‐fit test, calibration belt plot, and the Akaike Information Criterion (AIC) and Bayesian Information Criterion (BIC) values. An area under the receiver operating characteristic curve (AUROC) value above 0.5 signifies the model's capacity to differentiate between the two groups, while a lower *p* value further validates its discriminative ability [18, 19]. A Hosmer−Lemeshow test *p* value above 0.05 indicates that the model corresponds well with the observed data, signifying a good fit. Furthermore, sensitivity and specificity measures were used to evaluate the model's predictive accuracy [20]. AIC and BIC values were utilized to evaluate the trade‐off between model complexity and fit, where lower values signified better performance. These assessment metrics provided a thorough evaluation of the model's predictive and explanatory strength.

## 3 Results

**Figure 2:**
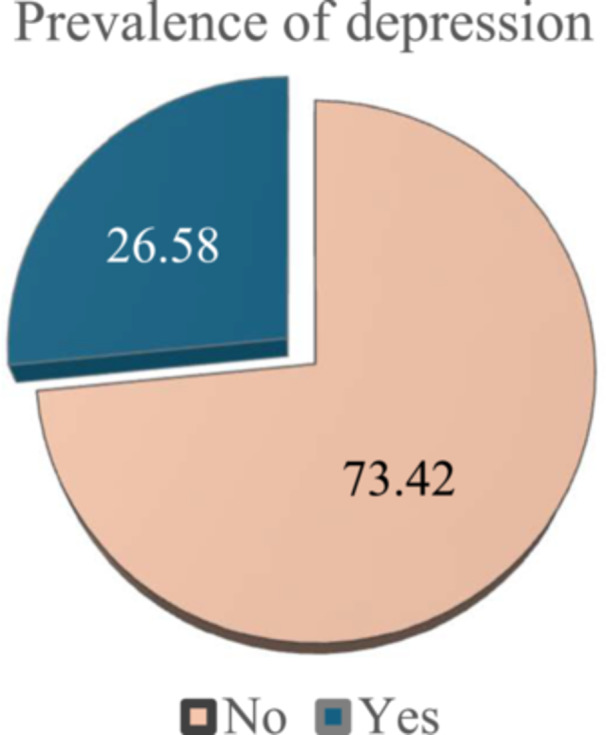
The prevalence of depression. Figure 2 illustrates the overall prevalence of depression among the study population. Approximately 26.6% of respondents reported experiencing depression (“Yes”), while 73.4% did not (“No”). The chart highlights that nearly one‐fourth of participants were affected by depression, reflecting a notable mental health concern within the population.

**Figure 3:**
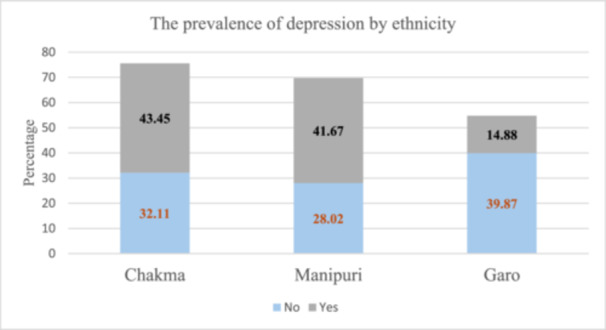
The prevalence of depression by ethnicity in Bangladesh. Figure 3 presents the distribution of depression among three ethnic groups in Bangladesh: Garo, Manipuri, and Chakma. The results show that the prevalence of depression was highest among the Chakma (43.45%), followed by Manipuri (41.67%), and lowest among the Garo (14.88%). The remaining proportions indicate individuals who did not experience depression within each group.

The study examines the association between depression and various socio‐demographic, economic, and lifestyle factors. Significant associations were found with age, ethnicity, religion, occupation, monthly hospital visits, wealth condition, care support, current health status, longstanding health problems, consultation time with doctors, meat consumption, fruit intake, sugar consumption, and alcohol use (*p *< 0.05). The mean age of participants was 59.73 years (SD = 8.08). Older individuals (≥ 71 years), certain ethnic groups (Chakma and Manipuri), and those with longstanding health problems had higher depression prevalence. Wealthier individuals and those consuming more sugar, alcohol, and red meat also showed higher depression rates. Other factors like gender, BMI, marital status, diabetes, hypertension, and sleep quality were not significantly associated with depression (*p* > 0.05) (Table 1). Among 632 participants (*N* = 632), the ethnic distribution was Chakma (35.13%, 222/632), Manipuri (31.65%, 200/632), and Garo (33.23%, 210/632). The prevalence of depression was highest among the Chakma group (43.45%, 73/632) and the Manipuri group (41.67%, 70/632), compared to the Garo group (14.88%, 25/632) (Figures 2 and 3).

**Table 1:** The frequency distribution of independent variables with depression.

Variables	Depression			
	No (%)	Yes (%)	Total (%)	*p* value
Gender				
Male	278 (59.91)	93 (55.36)	371 (58.70)	0.30
Female	186 (40.09)	75 (44.64)	261 (41.30)	
Age				
< 60 years	233 (50.22)	59 (35.12)	292 (46.20)	0.001*
61−70 years	194 (41.81)	80 (47.62)	274 (43.35)	
71+ years	37 (7.97)	29 (17.26)	66 (10.44)	
Ethnicity				
Chakma	149 (32.11)	73 (43.45)	222 (35.13)	0.001*
Manipuri	130 (28.02)	70 (41.67)	200 (31.65)	
Garo	185 (39.87)	25 (14.88)	210 (33.23)	
BMI				
Underweight	82 (17.75)	33 (19.64)	115 (18.25)	0.736
Normal	352 (76.19)	123 (73.21)	475 (75.40)	
Overweight	28 (6.06)	12 (7.14)	40 (6.35)	
Religion				
Islam	8 (1.72)	6 (3.57)	14 (2.22)	0.001***
Hindu	140 (30.17)	69 (41.07)	209 (33.07)	
Christian	187 (40.30)	26 (15.48)	213 (33.70)	
Buddhist	129 (27.80)	67 (39.88)	196 (31.01)	
Occupation				
Unemployment	122 (26.29)	57 (33.93)	179 (28.32)	0.002**
Business	63 (13.58)	7 (4.17)	70 (11.08)	
Small trader	29 (6.25)	14 (8.33)	43 (6.80)	
Job holder	24 (5.17)	10 (5.95)	34 (5.38)	
Labor	176 (37.93)	51 (30.36)	227 (35.92)	
Others	50 (10.78)	29 (17.26)	79 (12.5)	
Education				
Illiterate	293 (41.59)	77 (45.83)	270 (42.72)	0.21
Class 5	90 (19.40)	39 (23.21)	129 (20.41)	
Class10	38 (8.19)	22 (13.10)	93 (14.72)	
Class12	38 (8.19)	15 (8.93)	53 (8.39	
Can read and write, but attend school	72 (15.52)	15 (8.93)	87 (13.77)	
Marital status				
Married	408 (87.93)	147 (87.5)	555 (87.82)	0.88
Widow	56 (12.07)	21 (12.50)	77 (12.18)	
Monthly visit to hospital with family				
No	224 (48.28)	102 (60.71)	326 (51.58)	0.006**
Yes	240 (51.72)	66 (39.29)	306 (48.42)	
Income (BD TK)				
0−1000	337 (72.63)	129 (76.79)	466 (73.73)	0.069
11,000−15,000	87 (18.75)	19 (11.31)	106 (16.77)	
16,000−20,000	26 (5.60)	10 (5.95)	36 (5.70)	
21,000−25,000	14 (2.72)	5 (4.27	19 (3.01)	
26,000−30,000	4 (0.86)	1 (0.6)	5 (0.79	
Wealth condition				
Poorest	41 (8.84)	28 (16.67)	69 (10.92)	0.005**
Poorer	117 (25.22)	54 (32.14)	171 (27.06)	
Poor	135 (29.09)	32 (19.05)	167 (26.42)	
Medium	147 (31.68)	48 (28.57)	195 (30.85)	
Rich	24 (5.17)	6 (5.13)	30 (4.75)	
Care				
Wife	209 (45.04)	65 (38.69)	274 (43.35)	0.035***
Husband	27 (5.82)	8 (4.76)	37 (5.86)	
Daughter	63 (13.58)	16 (9.52)	79 (12.50)	
Son	73 (15.73)	44 (26.19)	117 (18.51)	
Self	92 (19.83)	35 (20.83)	127 (20.09)	
Financial support				
Son	258 (55.60)	113 (67.26)	371 (58.7)	0.11
Daughter	29 (6.25)	8 (4.76)	37 (5.85)	
Son/daughter in law	31 (6.68)	10 (5.95)	41 (6.49)	
Self	116 (25)	31 (18.45)	147 (23.26)	
Others	30 (6.47)	6 (3.57)	36 (5.7)	
Current situation				
Healthy	168 (36.21)	28 (16.67)	196 (31.01)	0.001*
Fairly healthy	208 (44.83)	72 (42.86)	280 (44.30)	
Moderate unhealthy	67 (14.44)	50 (29.76)	117 (18.51)	
Severe unhealthy	21 (4.53)	18 (10.71)	39 (6.17)	
Longstanding problems				
Yes	160 (34.48)	121 (72.02)	281 (44.46)	0.001*
No	304 (65.52)	41 (27.98)	351 (55.54)	
Diabetes				
No	376 (81.03	139 (82.74)	415 (81.49)	0.62
Yes	88 (18.97)	29 (17.26)	117 (18.51)	
Hypertension				
No	353 (76.08)	128 (76.19)	515 (81.49)	0.97
Yes	111 (23.92)	40 (23.81)	117 (18.51)	
Consulted health				
MBBS	296 (63.79)	101 (60.12)	397 (62.82)	0.72
Compounder	47 (10.13)	17 (10.12)	64 (10.13)	
Kabiraj	40 (8.62)	14 (8.33)	54 (8.54)	
Village doctor	81 (17.46)	36 (21.43)	117 (18.51)	
Talk to doctor				
< 15 min	415 (89.44)	140 (83.33)	555 (87.82)	0.038***
> 15 min	49 (10.56)	28 (16.67)	77 (12.18)	
Take meal				
2 serve/day	160 (34.48)	63 (37.50)	223 (35.28)	0.46
3 serve/day	259 (55.82)	85 (50.60)	344 (54.43)	
more than 3/day	45 (9.70)	20 (11.90)	65 (10.28)	
Meat weekly				
Yes	185 (39.87)	50 (29.94)	235 (37.24)	0.023***
No	279 (60.13)	117 (70.06)	396 (62.76)	
Red meat				
Yes	153 (32.97)	41 (24.55)	194 (30.74)	0.043*
No	311 (67.03)	126 (75.45)	437 (69.26)	
Egg				
< 3 serve/week	350 (75.43)	130 (77.38)	480 (75.95)	0.61
> 3 serve/week	114 (24.57)	38 (22.62)	152 (24.05)	
Fruit				
< 2/day	360 (77.75)	143 (85.63)	503 (79.84)	0.03***
> 2/day	103 (22.25)	24 (14.37)	127 (20.16)	
Vegetables				
< 2 serve/day	109 (23.49)	49 (29.17)	158 (25)	0.146
> 2 serve/day	355 (76.51)	119 (70.83)	474 (75)	
Sugar				
No	128 (26.94)	79 (47.02)	204 (32.28)	0.001*
Low	210 (45.26)	62 (36.96)	272 (43.04)	
Medium	129 (27.80)	27 (16.07)	156 (24.68)	
Earlier used sugar				
Low	295 (63.58)	117 (69.64)	412 (65.19)	0.15
Medium	169 (36.42)	51 (30.36)	220 (34.81)	
Use of oil				
Palm	55 (11.85)	20 (11.90)	75 (11.87)	0.93
Soyabean	372 (80.17)	137 (81.55)	509 (80.54)	
Rich bran	15 (3.23)	4 (2.38)	19 (3.01)	
Mustard oil	22 (4.74)	7 (4.17)	29 (4.59)	
Soft drink				
Yes	123 (26.51)	47 (27.98)	170 (26.90)	0.71
No	341 (73.49)	121 (72.02)	462 (73.10)	
Milk production				
Yes	98 (21.12)	26 (15.48)	124 (19.62)	0.11
No	366 (78.88)	142 (84.52)	508 (80.38)	
Alcohol				
Yes	168 (36.21)	37 (22.02)	205 (32.44)	0.001*
No	296 (63.79)	131 (77.98)	427 (67.56)	
Sleep quality				
No	151 (32.54)	65 (38.69)	216 (34.18)	0.150
Yes	342 (66.41)	103 (61.31)	416 (65.82)	
Outside activity				
Yes	247 (53.23)	86 (51.19)	333 (52.69)	0.65
No	217 (46.77)	82 (48.81)	299 (47.31)	

*Note:* (Significance level: *p* < 0.001*, *p* < 0.01** & *p* < 0.05***).

The adjusted logistic regression model identifies significant predictors of depression. Individuals aged 71+ years had 2.85 times higher odds of having depression compared to those under 60 (*p* < 0.05). Ethnicity played a strong role, with Chakma individuals having 3.19 times higher odds and Manipuri individuals having 9.16 times higher odds compared to the Garo group (*p* < 0.01). Those with longstanding health problems were 6.47 times more likely to have depression (*p* < 0.01). Consulting with a doctor for less than 15 min was associated with a 3.20 times higher risk (*p* < 0.01). Additionally, individuals with hypertension had 1.68 times higher odds of depression compared to those without (*p* < 0.05). These findings highlight the influence of age, ethnicity, chronic health conditions, doctor consultations, and hypertension on depression (Table 2).

**Table 2:** Outcome of adjusted logistic regression model.

Variables	Adjusted model		
	Odds ratio	95% CI	*p* value
Age			
Less than 60	Reference		
60−70	1.25	0.75−2.52	0.30
71+	2.85	1.19−5.96	0.01**
Ethnicity			
Garo	Reference		
Chakma	3.19	1.59−6.41	0.001*
Manipuri	9.16	4.8−17.47	0.001*
Long‐standing problem			
No	Reference		
Yes	6.47	3.93−10.66	0.001*
Talk with Doctor			
< 15 min	Reference		
> 15 min	3.20	1.58−6.47	0.001*
Hypertension			
No	Reference		
Yes	1.68	1.01−2.811	0.044***

*Note:* (Significance level: *p* < 0.001*, *p* < 0.01** & *p* < 0.05***).

**Figure 4:**
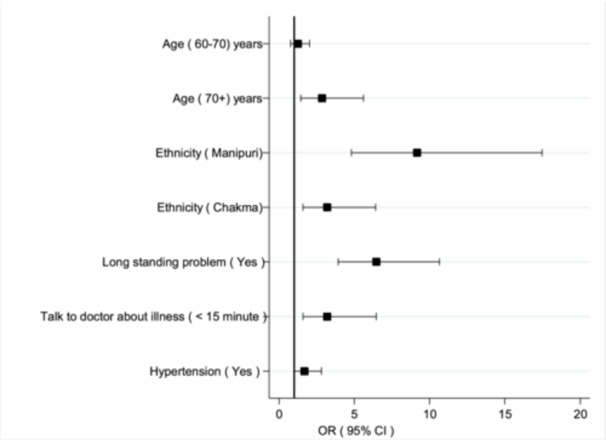
The forest plot of the logistic regression model. The adjusted multivariable logistic regression model shows significant predictors of the outcome in Figure 4. The results describe that older age (≥ 71 years: OR = 2.85, 95% CI: 1.19–5.96, *p* = 0.01), ethnicity (Chakma: OR = 3.19, 95% CI: 1.59–6.41, *p* < 0.001; Manipuri: OR = 9.16, 95% CI: 4.80–17.47, *p* < 0.001), having a long‐standing problem (OR = 6.47, 95% CI: 3.93–10.66, *p* < 0.001), longer consultation time (> 15 min: OR = 3.20, 95% CI: 1.58–6.47, *p* < 0.001), and hypertension (OR = 1.68, 95% CI: 1.01–2.81, *p* = 0.044) were significantly associated with the outcome.

**Figure 5:**
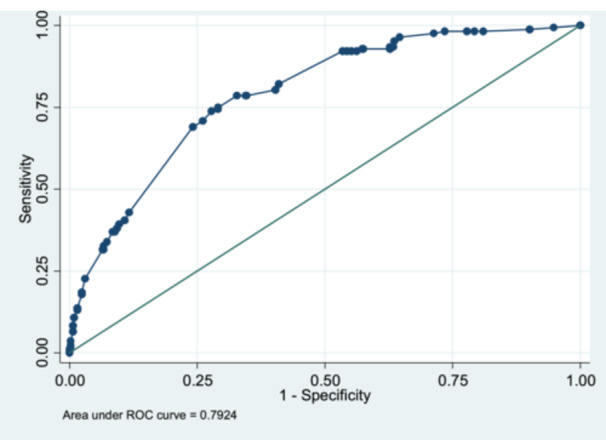
The area under ROC curve of logistic regression model. Figure 5 shows the goodness‐of‐fit statistics for the multivariable logistic regression model. The model demonstrated good fit with low AIC (606.68) and BIC (642.27), strong discrimination (AUROC = 79.2%, 95% CI: 0.75–0.83), nonsignificant Hosmer–Lemeshow test (*χ*² = 3.74, *p* = 0.809), and overall classification accuracy of 77.06%.

The goodness‐of‐fit statistics for the multivariable logistic regression model indicate strong model performance. The AIC and BIC values are 606.68 and 642.27, respectively, suggesting a well‐fitted model. The AUROC is 79.2% (95% CI: 0.75−0.83), demonstrating good discrimination ability. The Hosmer−Lemeshow test (*χ* ^2^ = 3.74, *p* = 0.809) confirms a good fit, as the high *p* value indicates no significant difference between observed and predicted values. Additionally, the model achieves a classification accuracy of 77.06%, further supporting its reliability (Table 3, Figures 4 and 5).

**Table 3:** Goodness of fit of the multivariable logistic regression model.

Goodness of fit of the multivariable logistic regression model				
AIC	BIC	AUROC (95% CI)	Hosmer and Lemeshow's goodness of fit	Classification accuracy
606.68	642.27	79.2% (0.75−0.83)	*χ* ^2 ^= 3.74, *p* = 0.809	77.06%

## 4 Discussion

This study found that approximately 27% of elderly participants reported depressive symptoms, with significant associations observed between depression and age, ethnicity, chronic health conditions, consultation with doctors, and hypertension. Individuals aged 71 years and above, those from the Manipuri and Chakma ethnic groups, and participants with longstanding health problems had a higher prevalence of depression. Interestingly, lifestyle factors such as higher sugar, alcohol, and red meat consumption were also associated with increased depressive symptoms, while gender, BMI, marital status, and diabetes showed no significant association.

The observed association between older age and depression is consistent with prior studies that report an increasing risk of depressive symptoms with advancing age [21]. A similar result of this study is that there is a strong positive relationship between depression and age groups. People aged 60−70 years are 1.25 times and 70+ years age is 2.85 times more likely to experience depression corresponding to less than 60 years age groups. Depression is more frequent among women, older adults, widowed or divorced individuals, and those with lower socioeconomic status. The prevalence is also higher in those lacking leisure‐time physical activity, having recent medical visits, or experiencing hospitalizations. After adjusting for confounders, depression was 1.44 times more common in individuals with one chronic disease and 2.25 times higher in those with two or more. For example, the strong relationship between chronic conditions and depression reflects evidence from earlier studies linking multiple comorbidities with higher depression prevalence. A greater burden of chronic diseases substantially increases the likelihood of depression [22]. In previous study found that chronic diseases such as depression 1.47 times, arthritis 2.14 times, asthma 3.36 times, chronic lung disease 3.74 times, angina 3.20 times, and stroke 3.14 times were significantly linked to depression (*p* < 0.001) [23].

Depression was linked to increased rates of cardiovascular diseases, type 2 diabetes, and metabolic syndrome. Exercise was key in preventing and treating these conditions, benefiting patients with both mental and physical health issues. It also improves body image, stress coping, quality of life, and independence in daily activities, particularly in older adults [24]. In this study, people with long‐standing health problems are about 6.5 times more likely to be depressed than those without such problems. Women who took their infants for three or more non‐routine doctor visits had a higher depression prevalence (32.6% vs. 13.6%, OR 2.87, *p* = 0.004). Those with fewer visits had a 58.3% lower relative risk (*p* = 0.002) and reported poorer family emotional support [25]. In the previous study in China, physical pain (16.2%) and poor sleep quality (20.0%) were significantly linked to doctor visits, while depression (14.4%) was not after adjustments. Screening for depression in older Chinese adults should focus on pain and sleep quality [26]. Higher life satisfaction was linked to fewer doctor visits. Each unit increase on a six‐point scale reduced visits by 11% (OR = 0.89, 95% CI 0.86–0.93). The most satisfied individuals (17.58%) had 44% fewer visits than the least satisfied (2.85%). This association persisted after adjusting for health and other factors (RR = 0.96, 95% CI 0.93–0.99), suggesting potential benefits for reducing healthcare costs [27].

Depression may impact health outcomes by affecting doctor–patient communication. Depressed patients (PHQ‐8 ≥ 10) were more likely (*p* < 0.05) to report suboptimal communication in five of seven domains, including clarity and patient‐centered decision‐making. Overall, 39.8% of depressed patients reported poor communication versus 22.9% of non‐depressed patients (*p* < 0.001). Adjusted analyses confirmed this association (OR 2.42, 95% CI 1.52–3.87, *p* < 0.001). Depressed patients with acute coronary syndrome (ACS) symptoms experienced poorer communication in the emergency department (ED), highlighting the need for research on objective differences [28].

Identified risk factors for depression in elderly hypertensive patients are modifiable, suggesting prevention through lifestyle changes and psychological education [29]. Depression is linked to increased morbidity, reduced quality of life, and higher mortality in hypertensive patients. Identifying and addressing depression in this population is crucial for improving overall health outcomes [30, 31]. Support vector machine (SVM) effectively distinguishes hypertensive patients with and without depression, revealing significant associations with blood tests and vital signs. This approach shows promise for clinical depression diagnosis, but further research is needed to validate these potential markers [32].

Depression affects people of all backgrounds, with around 18 million Americans experiencing mood disorders, including 10 million with major depression. Two‐thirds go untreated. This multifactorial disease stems from genetic, psychosocial, and environmental factors and often coexists with other conditions. MDD is widespread but frequently underdiagnosed, especially among minorities. Studies show African Americans facing socioeconomic stress are less likely to report symptoms or adhere to treatment. While minorities experience fewer acute MDD episodes than Caucasians, they are more prone to chronic, debilitating depression, severely impacting daily life [33].

Depression during pregnancy was more prevalent among ethnic minorities from the Middle East and South Asia. Crude prevalence rates were: Western Europeans 8.6%, Middle Easterners 19.5%, South Asians 17.5%, and other groups 11.3%. The median Edinburgh Postnatal Depression Scale (EPDS) score was 6 for Middle Easterners and 3 for others [34]. Ethnicity plays a key role in the link between social support and depression in the elderly. This study of 493 seniors in northern Chile (147 Aymara and 346 nonindigenous) found significant differences by sex, ethnicity, and marital status. Family was central in both groups, but structural, functional, and community support influenced depression differently across ethnicities [35]. Our findings that the Manipuri and Chakma groups were at greater risk align with previous research indicating that ethnicity and minority status play an important role in shaping mental health outcomes. This study aligns with previous findings, showing that the Manipuri ethnic group is 9.16 times more likely to experience depression. In contrast, the Chakma group is 3.19 times more likely, compared to the Garo ethnic group.

These findings highlight the importance of incorporating routine depression screening, such as PHQ‐9, into community health programs for older adults in Bangladesh. The higher risk among ethnic minorities suggests that culturally tailored interventions are needed to address unique social and cultural determinants of depression. Furthermore, the associations with dietary habits and chronic illness underscore the need for integrated approaches combining mental healthcare with lifestyle modification and chronic disease management.

This study used the PHQ‐9 as a screening tool to identify the presence of depressive symptoms within the study population. As the PHQ‐9 does not provide a clinical diagnosis, our findings should be interpreted as indicative of symptom prevalence rather than confirmed cases of depressive disorder. Furthermore, since no mental health professionals were included among the study authors, the interpretation of results is limited from a clinical perspective. Future research would benefit from collaboration with mental health experts to strengthen diagnostic accuracy and contextual interpretation.

## 5 Limitations

This study has several limitations. First, depressive symptoms were assessed using the PHQ‐9, a self‐reported screening tool, which may be subject to reporting bias and cannot be considered equivalent to a clinical diagnosis. Second, the cross‐sectional design limits the ability to establish causal relationships between depression and associated factors. Third, the absence of mental health professionals among the study authors may have restricted the clinical interpretation of the findings. Finally, as the study was conducted in a specific population, the generalizability of the results to other settings may be limited. Despite these limitations, the study provides important insights into depressive symptoms in the target population and can serve as a basis for future research. Although purposive sampling was used to focus on older adults, this approach may introduce selection bias. However, the age restriction was intentional, as the study aimed to assess depression among older adults in diverse ethnic communities. Future research could employ random sampling across broader age ranges to improve generalizability.

## 6 Conclusion

This study underscores the significant impact of depression among older adults in Bangladesh, particularly within the Garo, Manipuri, and Chakma ethnic groups. The findings highlight a strong link between depression and factors such as age, ethnicity, chronic health conditions, doctor consultations, and hypertension. Notably, depression rates were significantly higher among the Manipuri and Chakma groups compared to the Garo, shedding light on the role of socio‐cultural and healthcare disparities in shaping mental health outcomes. Chronic health issues emerged as a key risk factor, emphasizing the urgent need for targeted interventions to improve access to healthcare and mental health support. To effectively address depression in aging indigenous communities, culturally sensitive approaches, improved healthcare accessibility, and preventive strategies are essential. Moving forward, research and policy efforts should focus on developing tailored mental health programs that promote well‐being and ensure equitable care for elderly individuals in Bangladesh's ethnic communities.

## Author Contributions

**Kanis Fatama Ferdushi:** conceptualized the study, designed the sampling strategy, conducted data collection, guided the data analysis, wrote, reviewed, and edited the manuscript, and supervised the study. **Al Mahmud:** performed the data analysis and wrote, reviewed, and edited the manuscript. **Sabina Islam:** reviewed and edited the manuscript. **S.M. Saydur Rahman:** reviewed and edited the manuscript. All authors have read and approved the final version of the manuscript.

## Ethics Statement

The Shahjalal University of Science and Technology (SUST) Research Center, Bangladesh [Project Number PS/2022/2/32] granted ethics approval, including participants' approach and recruitment. We contacted the Ward Councilors [representatives of the government administrative ward] of Sylhet City Corporation and provided them with a Letter of Introduction and an Information Sheet about the research. These Councilors organized ward‐based yard gatherings on alternative days, where the Information Sheet was read out to potential participants by the Councilors, as most of the older adults were illiterate. The Councilors also informed the older adults about the aims of the research and introduced the researchers at the end of all the gatherings. A written Informed Consent form approved by the SUST‐Research Center was used to obtain consent from each of the older adults who expressed their interest in participating in the study. The participants who were illiterate used their thumbprints to provide consent. Regarding reviewing medical records of the participants, we contacted the Sylhet Civil Surgeon [Main authority in providing and managing healthcare for people living in Sylhet district], who considered the project's ethics approval, participants' consents, and our application for accessing the participants' medical data sets.

## Conflicts of Interest

The authors declare no conflicts of interest.

## Transparency Statement

The lead author Kanis Fatama Ferdushi affirms that this manuscript is an honest, accurate, and transparent account of the study being reported; that no important aspects of the study have been omitted; and that any discrepancies from the study as planned (and, if relevant, registered) have been explained.

## Supporting information


**Table S1:** PHQ‐9 Depression Scale.

## Data Availability

The data sets generated and/or analyzed during the current study are available from the corresponding author.
