# Supplementary material for: Aging, Economic Hardship, and Depression Among Ethnic Groups in Bangladesh: A Cross‐Sectional Study
Source: Health Sci Rep. 2026 Jan 8;9(1):e71732. doi: 10.1002/hsr2.71732 (PMC12783213; doi:10.1002/hsr2.71732)
Supplement: Supplementary file 1 — Table S1: PHQ‐9 Depression Scale. [file HSR2-9-e71732-s001.docx]

**Table S1: PHQ-9 Depression Scale**

| **Over the last 2 weeks, how often have you been bothered by any of the following problems?** | NOT AT ALL | SEVERAL DAYS | MORE THAN HALF THE DAYS | NEARLY EVERY DAY |
| --- | --- | --- | --- | --- |
| 1. Little interest or pleasure in doing things | ○ 0 | ○ 1 | ○ 2 | ○ 3 |
| 1. Feeling down, depressed, or hopeless | ○ 0 | ○ 1 | ○ 2 | ○ 3 |
| 1. Trouble falling or staying asleep, or sleeping too much | ○ 0 | ○ 1 | ○ 2 | ○ 3 |
| 1. Feeling tired or having little energy | ○ 0 | ○ 1 | ○ 2 | ○ 3 |
| 1. Poor appetite or overeating | ○ 0 | ○ 1 | ○ 2 | ○ 3 |
| 1. Feeling bad about yourself – or that you are a failure or have let yourself or your family down | ○ 0 | ○ 1 | ○ 2 | ○ 3 |
| 1. Trouble concentrating on things, such as reading the newspaper or watching television | ○ 0 | ○ 1 | ○ 2 | ○ 3 |
| 1. Moving or speaking so slowly that other people could have noticed. Or the opposite – being so fidgety or restless that you have been moving around a lot more than usual | ○ 0 | ○ 1 | ○ 2 | ○ 3 |
| 1. Thoughts that you would be better off dead, or of hurting yourself in some way | ○ 0 | ○ 1 | ○ 2 | ○ 3 |

| If you checked off any problems on this questionnaire so far, how difficult have these problems made if for you to do your work, take care of things at home, or get along with other people? | | | |
| --- | --- | --- | --- |
| Not difficult at all  ○ 0 | Somewhat difficult  ○ 1 | Very difficult  ○ 2 | Extremely difficult  ○ 3 |

**SCORING:**

Each response from the PHQ9 has a score ranging from 0 to 3. The score for each response is next to the check box. After a patient has completed the PHQ9, add up each column score, and then sum all four columns for the patient’s score. Below are the scoring guidelines for the PHQ9.

**Scoring Guidelines**

| **Guidelines for Interpretation of PHQ9*** | | |
| --- | --- | --- |
| **Score** | **Risk Level** | **Intervention** |
| 0-4 | No to Low risk | None, rescreen annually |
| 5-9 | Mild | Watchful waiting; repeat PHQ9 at follow up |
| 10-14 | Moderately | Treatment plan, considering counseling, follow-up and/or pharmacotherapy |
| 15-19 | Moderately Severe | Active treatment with pharmacotherapy and/or psychotherapy |
| 20+ | Severe | Immediate initiation of pharmacotherapy and if, severe impairment or poor response to therapy, expedited referral to a mental health specialist for psychotherapy and/or collaborative management |

*Kroenke K, Spitzer RL. (2002). The PHQ-9: A new depression and diagnostic severity measure. *Psychiatric Annals*, 32, 509-521.

*NOTE: If the patient responds to question 9 with any answer other than “not at all,” a suicide risk assessment needs to be completed.*

*If the total score is 10 or more, this could indicate a clinically significant problem and should trigger referral to a mental health program or enrollment in the Mental Health Integration Program.*
